# Recent Advances in Improving Gene-Editing Specificity through CRISPR–Cas9 Nuclease Engineering

**DOI:** 10.3390/cells11142186

**Published:** 2022-07-13

**Authors:** Xiaoqiang Huang, Dongshan Yang, Jifeng Zhang, Jie Xu, Y. Eugene Chen

**Affiliations:** Center for Advanced Models for Translational Sciences and Therapeutics, University of Michigan Medical Center, Ann Arbor, MI 48109, USA; doyang@umich.edu (D.Y.); jifengz@umich.edu (J.Z.); jiex@umich.edu (J.X.)

**Keywords:** gene editing, CRISPR–Cas9, off-target, specificity, high fidelity, protein engineering

## Abstract

CRISPR–Cas9 is the state-of-the-art programmable genome-editing tool widely used in many areas. For safe therapeutic applications in clinical medicine, its off-target effect must be dramatically minimized. In recent years, extensive studies have been conducted to improve the gene-editing specificity of the most popular CRISPR–Cas9 nucleases using different strategies. In this review, we summarize and discuss these strategies and achievements, with a major focus on improving the gene-editing specificity through Cas9 protein engineering.

## 1. Introduction

CRISPR–Cas9 is a powerful gene-editing tool and has a wide range of applications in biotechnology and medicine [1,2,3,4]. It is increasingly popular thanks to its simplicity, scalability, and affordability. Compared with previous gene-editing methods such as zinc finger nucleases (ZFNs) and transcription activator-like effector nucleases (TALENs) which both require the design and creation of a custom protein for each target DNA, it is much easier to use CRISPR–Cas9 by designing only a short guide RNA (gRNA) sequence.

CRISPR–Cas9 is a binary system composed of a nuclease (Cas9) that can cleave DNA and a gRNA that specifies the target loci for cleavage. If the CRISPR–Cas9 system binds at an unintended site, usually as a result of sequence homology or mismatch tolerance, it will create off-target double-strand breaks (DSBs), leading to nonspecific editing events. Off-target genetic modifications are widely observed and sometimes can disrupt the function of otherwise normal genes [5,6,7]. Therefore, for safety concerns, the off-target effects of CRISPR–Cas9 have to be addressed prior to the therapeutic applications. 

The off-target activities of CRISPR–Cas9 depend on multiple factors, including the inherent specificity and amount of Cas9 protein [5], the length, content, and structure of gRNA [8,9], the targeted cell type and cellular state [10,11], and the target site [6]. In the past several years, a variety of approaches have been proposed to reduce off-target editing, together with the methods for detecting off-target effects. In this review, we will not discuss off-target detection; readers can refer to recent reviews for an overview and comparison of these methods [12,13,14,15,16]. Instead, we focus on strategies and implementations reported to increase editing specificity. The strategies utilized for reducing off-target effects include Cas9 nuclease engineering [17,18,19,20,21,22,23,24,25,26,27,28,29,30,31,32,33], using natural Cas9 nucleases with high specificity [34,35], the utility of base editors [36,37,38] and prime editors [39,40], gRNA design optimization and/or modulation [8,9,41,42,43,44,45,46], control of Cas9′s activity via direct delivery (e.g., ribonucleoprotein (RNP) [20,47], virus-like particles [48,49,50,51], cell-penetrating peptide-mediated delivery [52], mRNA [53], and self-limiting circuits [54]), and combination with anti-CRISPR proteins or CRISPR inhibitors [55,56,57,58]. Among these strategies, direct or indirect engineering of Cas9 proteins represents the majority of efforts, evidenced by the relatively large number of studies toward this direction (Table 1). Below, we summarize and discuss recent advances in improving the specificity of CRISPR–Cas9-mediated gene editing through Cas9 protein engineering. 

## 2. Cas9 Engineering to Improve Gene-Editing Specificity

The reason why nature has not selected a highly precise Cas9 enzyme is not fully understood. Native CRISPR–Cas9 is an adaptive immune system of bacteria and archaea that functions in protection against virus and plasmid invasion [62,63]. Phages can, however, escape CRISPR immunity through mutations in the target region to prevent its recognition [64]. To adapt to phage evasion in this way, the native CRISPR–Cas9 system may evolve a balance between the mismatch tolerance and specificity to impede the viral immune evasion by tolerating a few nucleotide mismatches (e.g., mismatches at the protospacer adjacent motif (PAM) distal region [21,30]).

It was shown that the DNA binding specificity of a catalytically inactive Cas9 mutant was sufficiently high in *Escherichia coli*, yielding no detectable off-target transcriptional repression in the *E. coli* transcriptome [65]; this finding suggests that the wild-type (WT) Cas9 may not produce detectable off-target edits when it is appropriately devised to edit the *E. coli* transcriptome. In the perspective of gene editing-based therapy, however, there are high chances of off-target events when editing the mammalian genomes [5,6,7], probably because they are thousands of times larger than the bacterial genome, and the homologous sequences to the target, which are potential off-target sites, exist more frequently. Nevertheless, recent progress in the development of various high-fidelity Cas9 variants suggested that the DNA promiscuity of native Cas9 enzymes can be to some extent overcome by protein engineering or evolution.

### 2.1. Cas9 Engineering Strategies

Many strategies have been developed to generate high-fidelity Cas9 variants. These strategies can be roughly classified into nonrational, rational, or combined approaches. Typical nonrational strategies are directed evolution-based approaches, typically consisting of random mutagenesis followed by high-throughput screening. Rational methods use the structure and/or function information to guide Cas9 variant design, often through computational modeling of point mutations. The combined strategies may integrate directed evolution with structure-guided engineering. Table 1 summarizes these strategies and representative Cas9 variants.

### 2.2. SpCas9 Variants for Improving Gene-Editing Specificity

**Figure 1:**
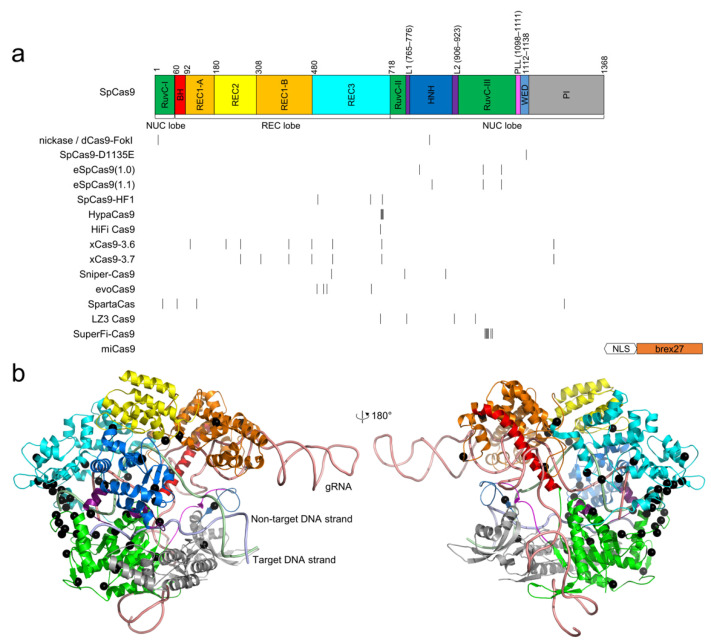
The locations of high-fidelity SpCas9 mutations. (**a**) domain organization of SpCas9. Notably, the domain architecture varies in different literature; the architecture by Huai et al. [67] is used. The positions of the mutated residues for all high-fidelity variants are indicated by black vertical lines. miCas9 contains the fused brex27 motif connected by the SV40 NLS linker instead of point mutations. (**b**) the point mutations, shown as black spheres, are mapped into the structure of SpCas9 (PDB ID: 5F9R) [68]. The color scheme for SpCas9 is identical to that in (**a**).

*Streptococcus pyogenes* Cas9 (SpCas9) is the most studied CRISPR–Cas9 nuclease for gene-editing applications, in part due to its short PAM requirement and high activity in eukaryotic cells. To address the issue that SpCas9 produces genome-wide off-target edits, researchers have developed a variety of high-fidelity SpCas9 variants. Table 2 summarizes the specific mutations of each high-fidelity SpCas9 variant. The locations of these mutations are highlighted in the primary and tertiary structure of SpCas9 (Figure 1). 

**Table 2:** Summary of SpCas9 variants for improving editing specificity.

Variants	Year	Mutations	References
SpCas9 nickase ^1^	2013	D10A or H840A	[25]
FokI-dCas9 ^1^	2014	D10A, H840A	[33,66]
SpCas9-D1135E	2015	D1135E	[27]
eSpCas9(1.0)	2016	K810A, K1003A, R1060A	[18]
eSpCas9(1.1)	2016	K848A, K1003A, R1060A	[18]
SpCas9-HF1	2016	N497A, R661A, Q695A, Q926A	[26]
HypaCas9	2017	N692A, M694A, Q695A, H698A	[21]
HiFi Cas9	2018	R691A	[20]
xCas9-3.6	2018	E108G, S217A, A262T, S409I, E480K, E543D, M694I, E1219V	[30]
xCas9-3.7	2018	A262T, R324L, S409I, E480K, E543D, M694I, E1219V	[30]
Sniper-Cas9	2018	F539S, M763I, K890N	[23]
evoCas9	2018	M495V, Y515N, K526E, R661Q	[19]
SpartaCas	2020	D23A, T67L, Y128V, D1251G	[24]
LZ3 Cas9 ^2^	2020	N690C, T769I, G915M, N980K	[29]
miCas9 ^3^	2020	The brex27 motif fused to SpCas9	[32]
SuperFi-Cas9	2022	Y1010D, Y1013D, Y1016D, V1018D, R1019D, Q1027D, K1031D	[28]

^1^ For SpCas9 nickase and FokI-dCas9, the Cas9 variants are used in pairs to increase gene-editing specificity. ^2^ The amino acid substitutions in LZ3 Cas9 are not explicitly described in the original paper by Schmid-Burgk et al. We collected the mutations from the deposited plasmid (https://www.addgene.org/140561/) (accessed on 8 June 2022). ^3^ miCas9 improves gene-editing specificity not through reducing non-specific recognition between gRNA and target DNA site but rather through enhanced homology-directed repair.

#### 2.2.1. SpCas9 Nickase

Cas9 nucleases contain two catalytic domains displaying cleavage activity, i.e., the HNH domain and the RuvC domain, respectively. A Cas9 nickase has one active and inactive nuclease domain and can only perform single-strand cleavage. Both domains can be mutated independently to generate Cas9 nickases. Nickases can be used in pairs to create DSBs in which each nickase cuts an opposite strand of the double-stranded DNA. In this strategy, a pair of SpCas9 nickases have to co-localize to bind and cut the target which substantially reduces off-target cleavage events compared to the WT SpCas9 [25].

#### 2.2.2. dCas9-FokI

Similar to the design of the nickase approach, dCas9-FokI is composed of a deactivated Cas9 (dCas9) fused to the FokI nuclease. Because the FokI nuclease only cleaves DNA upon dimerization activation, dCas9-FokI thus also works in pairs. The deactivation of Cas9 does not affect its binding to gRNA and target DNA. The gRNA guides the dCas9 to the target site and FokI will make the cleavage. The dCas9-FokI cleavage activity depends strictly on the binding of two gRNAs, substantially reducing the likelihood of off-target binding and thus increasing the cleavage specificity relative to WT SpCas9 [33,66]. However, for dCas9-FokI and SpCas9 nickase, the target sites are remarkably limited in the genome since they need to be dimer or paired for DSBs. In addition, the spacer between the two gRNAs needs to be optimized.

#### 2.2.3. miCas9

Our lab recently developed miCas9 to improve SpCas9′s homology-directed repairing capacity by fusing a minimal motif of BRCA2 (brex27) consisting of 36 amino acids to SpCas9 [32,69]. miCas9 binds RAD51 through the fused brex27 motif, enriching RAD51 at the target loci. The mechanism of action (MOA) for miCas9 to improve specificity is not through reducing non-specific binding, but rather through enhanced homology-directed repair such that the overall undesirable edits (e.g., off-target indels) are reduced [32]. As its MOA suggests, the miCas9 strategy is synergistic with other specificity-improving Cas9 variants (e.g., HiFi Cas9) [32]. In our ongoing studies, we found that the miCas9 fusion strategy also works to improve other Cas9 nucleases such as *Staphylococcus aureus* Cas9 (unpublished data).

#### 2.2.4. SpCas9-D1135E

While engineering SpCas9 nucleases with altered PAM specificities, Kleinstiver et al. unexpectedly discovered in 2015 that a D1135E mutant could yield a generalized improvement in genome-wide specificity relative to WT SpCas9, evidenced by GUIDE-seq experiments with three gRNAs on 25 previously known off-target sites [27]. Though not systematic, this observation refers to the first study showing that point mutation(s) can increase the genome-wide specificity of SpCas9.

#### 2.2.5. eSpCas9

By analyzing the SpCas9 structure, Slaymaker et al. proposed a model in 2016 that off-target cleavage occurred when the strength of Cas9 binding to the non-target strand exceeds that of DNA rehybridization [18]. Following this idea, they designed a variety of alanine-scanning mutations to weaken the interactions between Cas9 and the non-target strand. By combining several alanine substitutions, they developed “enhanced specificity” SpCas9 (eSpCas9), which represents the first systematic study to improve the specificity of SpCas9 through structure-based engineering.

#### 2.2.6. SpCas9-HF1, -HF2, -HF3, and -HF4

Almost at the same time as the development of eSpCas9, Kleinstiver et al. developed SpCas9-HF1, a high-fidelity variant harboring alterations designed to reduce non-specific DNA contacts, based on an “excess-energy” model [26]. In an earlier work, they proposed that the SpCas9-gRNA complex might possess more energy than is required for optimal recognition of its target DNA strand, thereby enabling cleavage of mismatched sites [8]. Thus, as opposed to the energy model for designing eSpCas9 [18], they reasoned that off-target effects of SpCas9 can be minimized by decreasing non-specific interactions with its target DNA strand [26]. By analyzing the experimental SpCas9 structure [70,71], they identified four SpCas9 residues (N497, R661, Q695, Q926) that are in direct contact with the backbone of the target DNA strand. They showed that SpCas9-HF1 (a quadruple mutant composed of N497A, R661A, Q695A, and Q926A) reduced all or nearly all genome-wide off-target effects to undetectable levels as judged by GUIDE-seq and targeted next-generation sequencing. They also combined SpCas9-HF1 with other point mutations to create SpCas9-HF2 (HF1 + D1135E), -HF3 (HF1 + L169A), and -HF4 (HF1 + Y450A). Data showed that SpCas9-HF2, -HF3, and -HF4 could further reduce indel frequencies at some residual off-target sites that persist for SpCas9-HF1 [26].

#### 2.2.7. HypaCas9

Both eSpCas9 and SpCas9-HF1 were developed based on an energy model that the SpCas9-gRNA complex possesses “excess energy” to bind DNA either through nonspecific interactions with either the nontarget or target strand; it was speculated that disabling such interactions would decrease the “excess energy” and reduce off-targets. The “excess-energy” model, however, was challenged by the biochemical studies by Chen et al., where they found that the affinities of the eSpCas9(1.1) and SpCas9-HF1 variants for both on-target and PAM-distal mismatched substrates were similar to that of the WT SpCas9 [21]. Through single-molecule Förster resonance energy transfer experiments, they showed that, when bound to mismatched targets, eSpCas9(1.1) and SpCas9-HF1 were trapped in an inactive state with their HNH domains populated in a catalytically inactive conformational checkpoint [21]. They found that the noncatalytic REC3 domain of SpCas9 senses target complementarity and indirectly govern the HNH domain to regulate the cleavage activation. Specifically, they proposed that REC3, when bound to the gRNA:DNA duplex, will re-orient REC2, which directly regulates HNH docking into the active state [21]. Based on this model, they identified five clusters of residues containing conserved amino acids within 5 Å of the gRNA–DNA interface, four of which are located within REC3 and one in the HNH–RuvC Linker 2, and mutated all the residues in them to alanines. GUIDE-seq experiments revealed that cluster 1, i.e., HypaCas9, significantly suppressed genome-wide off-target cleavage compared with WT SpCas9 and showed equivalent genome-wide specificity relative to eSpCas9(1.1) and SpCas9-HF1 [21].

#### 2.2.8. xCas9

In one of the first directed evolution attempts, Hu et al. used phage-assisted continuous evolution to evolve SpCas9 and obtained xCas9 variants with expanded PAM compatibility [30]. Although the primary goal of their study was not to increase the cleavage specificity, they unexpectedly found that xCas9-3.6 and -3.7 displayed much lower off-target activity than SpCas9 through GUIDE-seq analysis [30]. This finding challenged the conventional thought that the broadened PAM compatibility of Cas9 nucleases would increase their off-target activity and demonstrated that relaxing PAM requirements and increasing cleavage specificity may be achieved at the same time.

#### 2.2.9. evoCas9

In yet another directed evolution work to develop SpCas9 with enhanced specificity, Casini et al. developed a yeast-based assay that enables simultaneous evaluation of on- and off-target activity [19]. After a single round of directed evolution screening on a library of REC3 mutants, they initially isolated a shortlist of 14 SpCas9 variants with increased specificity, including variant C13, which adopts a single point mutation K526E. Then, they selected a set of mutations located close to the gRNA:DNA duplex and combined them with the K526E mutation. The best mutant, which contained the M495V/Y515N/K526E/R661L (VNEL) substitutions, displayed high specificity at the expense of ~20% drop in on-target activity. To rescue the editing efficiency, they rationally mutated L661 into glutamine (Q), which exhibited similar activity to WT SpCas9′s. The genome-wide off-target activity of this new variant, denoted as evoCas9, was evaluated using GUIDE-seq in a head-to-head comparison with eSpCas9(1.1), SpCas9-HF1, and SpCas9, with eight gRNAs targeting eight different loci. Their experimental data showed that, overall, evoCas9 showed the highest reduction in the total number of detected off-target sites [19]. 

#### 2.2.10. HiFi Cas9

One issue of the high-fidelity SpCas9 variants is that they have reduced on-target activity. To retain high on-target activity as well as reduce off-target activity, Vakulskas et al. developed an unbiased bacterial screening method to identify desired variants in the RNP format [20]. They found that a variant with a single-point mutation R691A (known as HiFi Cas9) retained the high on-target activity of SpCas9 while reducing off-target cleavage. It is worth mentioning that this mutation is in the immediate vicinity of residue N692, which is mutated into alanine in HypaCas9 (see Table 2). Five Cas9 RNPs, including HiFi Cas9, eSpCas9 (1.1), SpCas9-HF1, HypaCas9, and WT SpCas9, were tested in HEK293 cells targeting 12 sites within the HPRT locus. The median on-target activity (normalized to that of WT SpCas9), as indicated by indel frequency determined by next-generation sequencing (NGS), for each variant was 82% for HiFi Cas9, 20% for eSpCas9 (1.1), 2% for SpCas9-HF1, and 1.7% for HypaCas9 [20]. The data revealed that these multipoint mutagenic high-fidelity SpCas9 mutants might be overengineered to increase their editing specificity; the fact of their relatively low on-target activity (compared to WT SpCas9) may be covered up when delivered as overexpression plasmids [20].

#### 2.2.11. Sniper-Cas9

To improve SpCas9′s specificity without attenuating its cleavage activity, Lee et al. developed an *E. coli*-based directed evolution method, Sniper screen, to isolate SpCas9 variants with high specificity and activity [23]. The screening system allows simultaneous positive and negative selection for SpCas9 variants with high specificity without killing the on-target activity. Multiplex Digenome-seq analysis with four gRNAs showed that Sniper-Cas9 displayed much lower than WT-level off-target effects at all sites and did not cleave additional off-target sites compared with SpCas9 [23]. When compared with other engineered high-fidelity SpCas9 variants such as SpCas9-HF1, HypaCas9, evoCas9, and eSpCas9(1.1), Sniper-Cas9 showed much higher on-target activities and comparable specificity at most off-target loci. However, Sniper-Cas9 showed stronger tolerance to single mismatches at the PAM-distal region (e.g., 16th, 18th, and 19th) [23].

#### 2.2.12. SpartaCas

Similar to the development of Sniper-Cas9, Cerchione et al. developed another directed evolution strategy to screen SpCas9 variants with low off-target activities while retaining strong on-target editing [24]. Specifically, they devised a mutagenesis method termed scanning mutagenesis of oligo-directed targets for creating highly diverse libraries of SpCas9 variants followed by high-throughput M13 bacteriophage-mediated selection. The mutant, SpartaCas (*S. pyogenes* Adapted to Reduce Target Ambiguity Cas9), which is composed of the most enriched point mutations, was reported to have reduced off-target events while maintaining high on-target editing in T-cells [24].

#### 2.2.13. LZ3 Cas9

Schmid-Burgk et al. developed a rapid pipeline termed tagmentation-based tag integration site sequencing (TTISS) for analyzing double-strand cleavage events [29]. Using TTISS, they compared eight high-fidelity SpCas9 variants and the native SpCas9, revealing overall a tradeoff between cleavage specificity and activity. To investigate whether this tradeoff is a general feature, they carried out saturation mutagenesis on 157 residues in the HNH and RuvC domains and the L1 and L2 linkers connecting them (Figure 1). Next, they combined the top point mutations that exhibited both high on-target efficiency and high specificity to produce combinatorial mutants, including LZ3 Cas9, which showed similarly high on-target activity but significantly enhanced specificity relative to the native SpCas9 [29].

#### 2.2.14. SuperFi-Cas9

To explore the molecular basis by which Cas9 recognizes mismatches, very recently, Bravo et al. used kinetics-guided cryo-electron microscopy to solve the structure of SpCas9 at different phases of mismatch cleavage [28]. They observed that substrates with mismatches at the PAM distal region (i.e., 18th–20th) were stabilized by a reorganized loop (approximately amino acids 1009–1031) in the RuvC domain. Notably, the residues in this loop are usually missed in previous structures due to poor electron density. These findings suggested that this loop may play a critical role in mismatch recognition (especially at the PAM-distal part) but not in on-target activation. Per this hypothesis, the authors designed a 7D mutant (known as SuperFi-Cas9) by mutating the seven mismatch-stabilizing residues into aspartic acid. The competition assay showed that SuperFi-Cas9 displayed a 6.3-fold preference for on-target DNA relative to DNA bearing 18–20 mismatches, while WT SpCas9 showed only a low preference ratio of 1.55 fold [28]; this result indicated that SuperFi-Cas9 possessed a good ability to discriminate between on- and off-target substrates. A recent study (in the preprinted form) from an independent lab showed that SupperFi-Cas9 showed high-fidelity but significantly reduced on-target activity in mammalian cells [72].

#### 2.2.15. Other SpCas9 Variants

As WT SpCas9 requires a 5′-NGG-3′ PAM for target recognition which roughly limits its targeting range to 1/8 of the whole genome, researchers also try to create novel SpCas9 variants with altered or expanded PAM requirements. Numerous successful PAM altered or relaxed mutations were reported [27,30,73,74], and most of them, if not all, were found to be compatible with the high-fidelity mutations to simultaneously increase targeting range as well as reduce off-target edits. In addition, it was shown that low-copy expression of split-Cas9 fragments could be used for gene editing without high mutation at off-target sites [61]. SpCas9 chimeras by fusing active SpCas9 with other proteins/domains (e.g., programmable DNA-binding domain [59], dead or active Cas9 from either *N. meningiditis* or *S. aureus* [60]) also showed improved gene-editing specificity.

### 2.3. SaCas9 Variants for Improving Gene-Editing Specificity

**Figure 2:**
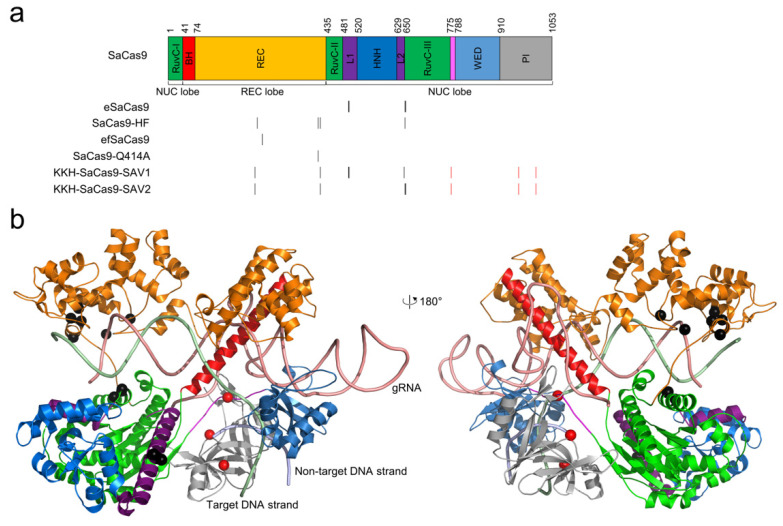
The locations of high-fidelity SaCas9 mutations. (**a**) domain organization of SaCas9. The architecture by Nishimasu et al. [79] is used. The positions of the mutated residues for all high-fidelity variants are indicated by black vertical lines. The PAM-relaxed KKH mutations are shown in red vertical lines. (**b**) the high-fidelity point mutations, shown as black spheres, are mapped onto the structure of SaCas9 (PDB ID: 5AXW) [79]. The KKH mutations are shown as red spheres. The color scheme for the structure is identical to that for the sequence.

In addition to the high-fidelity SpCas9 variants, other Cas9 proteins, such as *Staphylococcus aureus* Cas9 (SaCas9), with different PAM requirements, were isolated. SaCas9 is a good alternative to SpCas9 for gene-editing applications owing to its comparably high activity in eukaryotic cells [75,76,77]. In addition, SaCas9 has a more compact size (1053 amino acids for SaCas9 versus 1368 amino acids for SpCas9), making it easier to package into the payload-limited adeno-associated viral vector for in vivo gene editing [78]. Similar to SpCas9, SaCas9 also shows genome-wide off-target activity, limiting its practical application. Table 3 summarizes the high-fidelity SaCas9 variants reported so far. The positions of these mutations are mapped in the primary and tertiary structure of SaCas9 (Figure 2).

#### 2.3.1. eSaCas9

The first high-fidelity SaCas9 variant, eSaCas9, was reported by Slaymaker et al. as a sister variant to eSpCas9 [18]; both variants were created following the same model. eSaCas9 showed reduced activity at three predefined off-target sites to EMX site 7, but its genome-wide specificity has not been assessed.

#### 2.3.2. SaCas9-HF

Tan et al. designed SaCas9-HF following the same strategy as Kleinstiver et al. developed SpCas9-HF1, i.e., by reducing nonspecific interactions with the target DNA strand [22]. Based on the structure of SaCas9, they identified four amino acids (R245, N413, N419, and R654) that form hydrogen bonds with the backbone of the target DNA strand. They mutated each of them into alanine to investigate the variant’s activity and specificity at selected targets, and they found that the combination of four mutations (known as SaCas9-HF) yield the highest editing specificity. Genome-wide targeting specificity of SaCas9-HF evaluated by GUIDE-seq on 11 endogenous sites (six canonical and five noncanonical PAMs) showed that SaCas9-HF significantly reduced off-target activities and increased on- to off-target edit ratios compared to WT SaCas9 [22]. Assessment of an additional list of 13 targets showed that both SaCas9-HF and WT SaCas9 displayed no or minimal off-target events on seven sites while in the other six sites SaCas9-HF significantly exhibited reduced off-target activities (mean off-target site number 3.0 for SaCas9-HF vs. 9.8 for WT SaCas9, one-sided Mann–Whitney *U* test *p*-value = 0.039) [22].

#### 2.3.3. efSaCas9 and SaCas9-Q414A

Xie et al. used a directional screening system to identify Cas9 variants with desired properties, and with this system, they isolated efSaCas9 (enhance-fidelity SaCas9) which bears a single point mutation N260D [17]. Targeted deep-sequencing experiments showed that efSaCas9 substantially reduced off-target events compared to WT SaCas9. In addition, primer extension-mediated sequencing (PEM-seq) experiments showed that efSaCas9 possesses higher fidelity than WT SaCas9 at the genome-wide level while maintaining WT-like on-target activity. To gain structural insights into the enhanced fidelity of efSaCas9, they analyzed the structure of SaCas9 and found that N260 bridges a network of interactions that may affect SaCas9’s specificity. The authors made single-point mutations to three residues (Y256, Q414, and N419) around N260 and found that Q414A displayed even higher fidelity than N260D while retaining most on-target activity, as reported by enhanced green fluorescence protein (EGFP) disruption experiments targeting EGFP site 3 (PM3) [17]. However, the genome-wide specificity of SaCas9-Q414A was not evaluated.

#### 2.3.4. KKH-SaCas9-SAV1 and -SAV2

Yuen et al. used structure-guided mutagenesis to engineer a PAM-expanded version of SaCas9, i.e., KKH-SaCas9, with low off-target and high on-target activities [31]. Building on structural analysis, they initially mutated 12 residues that were predicted to be in direct contact with or close to the DNA or gRNA backbones to specified substitutions. Then, they generated and evaluated a library of 27 variants (i.e., v3.1–20, v3.22–25, and v3.27–29) randomly sampled from the combination of these substitutions together with the KKH mutations. Among them, GFP disruption experiments showed that KKH-SaCas9-SAV1 (v3.16 with mutations Y239H, N419D, R499A, Q500A, and Y651H) generated the least off-target events after a 15-day post-transduction while maintaining approximately 70% of on-target activity relative to KKH-SaCas9. Another variant, KKH-SaCas9-SAV2 (v3.10 bearing mutations Y239H, N419D, R654A, and G655A) showed slightly higher off-target activities than did KKH-SaCas9-SAV1 (but significantly lower than did KKH-SaCas9) and retained about 80% of the on-target activity compared to KKH-SaCas9. GUIDE-seq experiments showed that KKH-SaCas9-SAV1 and -SAV2 showed higher on- to off-targeting ratios than KKH-SaCas9 in all tested loci [31].

#### 2.3.5. Other SaCas9 Variants

The above high-fidelity SaCas9 variants were directly derived from the WT SaCas9 or KKH-SaCas9 [80]. Compared with SpCas9 which requires a simple 5′-NGG-3′ PAM, WT SaCas9 requires a long, noncanonical 5′-NNGRRT-3′ PAM sequence, further reducing the targeting range by 1/4. To overcome this limitation, a variety of studies were reported to design PAM-altered or -relaxed SaCas9 mutations and couple them with high-fidelity mutations [80,81,82]. 

### 2.4. Other Cas9 Proteins and Variants with High Gene-Editing Specificity

In addition to SpCas9 and SaCas9, many other Cas9 proteins (e.g., ScCas9 [83], CjCas9 [84], NmeCas9 [85], FnCas9 [86], SmacCas9 [87], SauriCas9 [88], St1Cas9 [89], St3Cas9 [90], FrCas9 [34], to name a few) have been isolated and some of them were reported to be intrinsically high-fidelity [34,35]. Some of them were engineered with a broad PAM range, high fidelity, and/or both [91].

## 3. Discussion, Conclusions, and Outlook

The fact that CRISPR–Cas9 gene-editing tools can result in genome-wide off-target edits may confound their application as therapeutics [5,6,7]. Therefore, reducing the off-target effects of Cas9 nuclease is a major goal to improve its precision for genome editing. As above reviewed, numerous strategies, especially protein engineering, have been utilized to improve the precision of Cas9 cleavage. Thanks to the rapid progress in this field, now we have a versatile toolbox of high-fidelity Cas9 proteins, including variants with point mutations, paired nickases, chimeric dCas9-FokI, fused miCas9, and other variants. To provide a guidance for readers to choose a Cas9 protein appropriate for their research, we summarize in Table 4 the biological systems in which the aforementioned high-fidelity SpCas9 and SaCas9 variants have been evaluated.

These engineered Cas9 variants have reduced off-target effects while maintaining the cleavage activity to different extents. Thus, choosing suitable Cas9 proteins or variants is critical for a specific gene-editing task. A systematic comparison of different Cas9 proteins to guide the selection is important and necessary, but challenging [107]. Recently, Schmid-Burgk et al. developed the TTISS protocol, a rapid and scalable method, for analyzing double-strand breaks [29]. With this method, they systematically compared the specificity and activity of eight high-fidelity SpCas9 variants. In another study, Kim et al. built 16 deep-learning models, collectively named DeepSpCas9Variants, to computationally predict the sequence-specific activity of 13 SpCas9 variants, including six high-fidelity variants, after assessing their cleavage efficiency at 26,891 target sequences [98]. The specificity-activity profile of the seven common variants (i.e., eSpCas9 (1.1), evoCas9, HypaCas9, SniperCas9, SpCas9-HF1, xCas9-3.7, and WT SpCas9) evaluated by DeepSpCas9Variants was similar to that by TTISS. Both methods suggested that evoCas9 had the highest specificity but lowest activity while WT SpCas9 was the opposite [29,98]. The DeepSpCas9Variants’ models would help assist with the choice of an appropriate SpCas9 variant for a target sequence in real-world applications. However, a limitation of DeepSpCas9Variants is that it only applies to current SpCas9 variants; for new SpCas9 variants, the models have to be retrained after evaluating their cleavage efficiency. In the future, similar predictive models may also be desirable for other Cas9 proteins including SaCas9.

Though a few high-fidelity Cas9 variants have been successfully harvested, they are far from perfect. For instance, the high-fidelity Cas9 variants are generated usually at the expense of a cleavage activity loss; however, the activity loss is largely covered up by the overexpression of engineered Cas9 variants when delivered as plasmids. It is shown that a lower dose of Cas9 can offer a greater specificity [48,50]. However, the inefficient high-fidelity Cas9 variants with transient delivery or in other formats (e.g., RNP) may hinder their therapeutic applications in vivo when the input dose is low. Thus, Cas9 variants with both high fidelity and activity are still being sought after. More importantly, the mechanism by which the Cas9 variants discriminate on- and off-target sites, as well as the machinery of off-target binding and activation, are not fully understood, due in part to the highly dynamic and complicated nature of Cas9 binding, recognition, and activation. A full picture of the on- and off-target discrimination would be extremely helpful in capturing the key factors which are critical in designing the next-generation high-fidelity Cas9 variants. In this regard, the recent kinetics-guided cryo-electron microscopy study of Cas9 nuclease in the presence of various gRNA:DNA mismatches strongly clued us in on the difference between on- and off-target binding and recognition [28]. However, in this structural biology study, only a single target site was investigated. As off-target editing is complex and target site-dependent [6], structural studies of more gRNA:DNA mismatch cases at different, nonhomologous target sites need further investigation.

## Figures and Tables

**Table 1:** Strategies for engineering high-fidelity Cas9 proteins and representative variants.

Classification	Strategy	Representative High-Fidelity Cas9 Variants
Nonrational	Directed evolution (random mutagenesis plus high-throughput screening)	Sniper-Cas9 [23], HiFi Cas9 [20], xCas9 [30], SpartaCas [24], efSaCas9 [17]
Rational	Structure- and/or function-guided protein engineering	SpCas9 nickase [25], SpCas9-D1135E [27], eSpCas9 [18], SpCas9-HF1 (and -HF2, -HF3, -HF4) [26], HypaCas9 [21], SuperFi-Cas9 [28], LZ3 Cas9 [29], eSaCas9 [18], SaCas9-HF [22], KKH-SaCas9-SAV1 (and -SAV2) [31]
	Fusion protein	dCas9-FokI [33], miCas9 [32], Cas9-pDBD [59], Cas-Cas9 chimeras [60]
	Protein splitting	split-Cas9 [61]
Combined	Directed evolution plus structure-guided modeling	evoCas9 [19], SaCas9-Q414A [17]

**Table 3:** Summary of SaCas9 variants for improving editing specificity.

Variants	Year	Mutations	References
eSaCas9	2016	R499A, Q500A, R654A, G655A	[18]
SaCas9-HF	2018	R245A, N413A, N419A, R654A	[22]
efSaCas9	2020	N260D	[17]
SaCas9-Q414A	2020	Q414A	[17]
KKH-SaCas9-SAV1	2022	Y239H, N419D, R499A, Q500A, Y651H (plus E782K, N968K, R1015H)	[31]
KKH-SaCas9-SAV2	2022	Y239H, N419D, R654A, G655A (plus E782K, N968K, R1015H)	[31]

**Table 4:** Summary of biological systems in which the high-fidelity SpCas9 and SaCas9 variants have been evaluated.

Variants	Evaluation Biological Systems
SpCas9 nickase	HEK293FT cells [25], HUES62 hES cells [25], human embryonic stem cells [5], human keratinocytes cells [92], mouse spermatogonial stem cells [93], mouse zygotes [25], rice [94]
FokI-dCas9	HEK293 cells [33], U2OS cells [33], HUES9 cells [66], chronic myeloid leukemia cells [95], HeLa cells [96], human induced pluripotent stem cells [96], mice [97]
SpCas9-D1135E	U2OS cells [27]
eSpCas9	HEK293 and HEK293T cells [18,29,98], rice [99,100], wheat [100]
SpCas9-HF1	U2OS cells [26], HEK293T cells [29,98], rice [100], wheat [100]
HypaCas9	U2OS cells [21], HEK293T cells [29,98], mouse zygotes [101], rice [99]
HiFi Cas9	human hematopoietic stem and progenitor cells [20], primary T cells [20], HEK293 cells [20,29,98], rice [102], marine algae [103]
xCas9	*E. coli* cells [30], HEK293T cells [29,30,98], U2OS cells [30], rice [104,105]
Sniper-Cas9	*E. coli* cells [23], HEK293T cells [23,29,98], HeLa cells [23], primary T cells [23], induced pluripotent stem cells [23]
evoCas9	Yeast cells [19], 293multiEGFP, 293blastEGFP, and HEK293T cells [19,29,98]
SpartaCas	T cells [24]
LZ3 Cas9	HEK293T cells [29], K562 cells [29], U2OS cells [29]
miCas9	induced pluripotent stem cells [32], airway epithelial cells [32], fibroblast cells [32], Jurkat cells [32], Ad293 cells [32]
SuperFi-Cas9	HEK293 cells [72], neuro-2a mouse neuroblastoma cells [72]
eSaCas9	HEK293 and HEK293T cells [18,22], human retinal pigmented epithelium cells [22]
SaCas9-HF	HEK293 and HEK293T cells [22,106], human retinal pigmented epithelium cells [22]
efSaCas9	HEK293 cells [17], HeLa cells [17], HT-1080 cells [17]
SaCas9-Q414A	HEK293 cells [17], HeLa cells [17], HT-1080 cells [17]
KKH-SaCas9-SAV1	HEK293T cells [31], SK-N-MC cells [31], MHCC97L cells [31], OVCAR8-ADR cells [31]
KKH-SaCas9-SAV2	HEK293T cells [31], SK-N-MC cells [31], MHCC97L cells [31], OVCAR8-ADR cells [31]

## Data Availability

Not applicable.
